# Total Phenolic and Flavonoid Contents of Aqueous Extract of *Stinging Nettle* and *In Vitro* Antiproliferative Effect on Hela and BT-474 Cell Lines

**Published:** 2014

**Authors:** Sadegh Fattahi, Ebrahim Zabihi, Zeinab Abedian, Roghayeh Pourbagher, Ali Motevalizadeh Ardekani, Amrollah Mostafazadeh, Haleh Akhavan-Niaki

**Affiliations:** 1 *Cellular and Molecular Biology Research Center, Babol University of Medical Sciences, Babol, Iran*.; 2 *National Institute of Genetic Engineering and Biotechnology (NIGEB), Tehran, Iran.*

**Keywords:** Polyphenols, *Stinging nettle*, antioxidant activity, antiproliferative effect

## Abstract

Phenolic compounds including flavonoids and phenolic acids are plants secondary metabolites. Due to their ability to act as antioxidant agents, there is a growing interest to use those components in traditional medicine for cancer prevention or treatment. The aim of this study was to measure the amounts of total phenolics and flavonoids as well as anti-proliferative effect of aqueous extract of *Stinging nettle* on BT-474 and Hela cell lines. The amounts of phenolics content and total flavonoids were determined by folin ciocalteu and aluminium chloride methods, respectively. The free radical scavenging activity was measured by using diphenyl - picrylhydrazyl (DPPH). The reducing power of the extract was measured in the presence of potassium hexacyanoferrate and its antiproliferative activity was assessed on BT-474 and Hela cell lines using MTT assay. Total phenolic content was 322.941± 11.811 mg gallic acid/g extract. Total flavonoid content was 133.916±12.006 mg Catechin/g. The IC50 of DPPH radical was 1.2 mg/ ml and the reducing power was 218.9± 15.582 μg ascorbic acid/ g. Cell viability of BT-474 cells decreased to less than half of the control (no added extract) at the presence of 3 mg/ ml extract while no significant changes were detected for Hela cells at similar conditions. There was no significant difference in the percentage of surviving cells between consecutive days (day 1, 2 and 3) for both BT-474 and Hela cells (P>0.05). Although the relatively high amount of phenolic and flavonoid contents of the aqueous extract make this plant a promising candidate for diseases treatment; however, there is not a direct relationship between the amounts of these antioxidant components and the efficiency in *in vitro* cancer treatment.

Tere has been an upsurge of interest in the use of plants, as a source in folk medicine to treat various chronic diseases. In western countries many prescriptions correspond to products originating from plants (1-3). Phenolic compounds are plant secondary metabolites possessing aromatic ring with one or more hydroxyl groups from the aromatic amino acids phenylalanine produced via the phenylpropanoid pathway (4). The two major classes of phenolic compounds include flavonoids and phenolic acids. The presence of phenolic compounds enables plants to act as reducing agents, hydrogen donators and singlet oxygen quenchers. During cell metabolism pathways, all living cells generate free radicals as part of normal cellular functions (5). Free radicals are highly reactive and may be either oxygen derived or nitrogen derived and can react with proteins, lipids, carbohydrates and DNA (6). An imbalance between reactive oxygen species and antioxidants may cause oxidative stress, leading to cellular damage and subsequently various diseases in man such as atherosclerosis, diabetes mellitus, arthritis, ischemia heart disease, gastritis, immunosuppression, neurodegenerative diseases, ageing and cancer (7-10). An inverse association between consumption of fruits and vegetables with lower risk of cancer is widely accepted and there have been great efforts in cancer treatment with their secondary metabolites (11-16). Therefore, the objective of this research was to measure the amounts of total phenolics, total flavonoids and anti-proliferative effect of aqueous extract of *Stinging nettle* on BT-474 and Hela cell lines.

## Materials and Methods

**Plant extract preparation**

An aqueous extract of leaves of *Stinging nettle* was obtained previously as described by Fattahi et al. (17). Briefly, 15 g of plant leaves were powdered and extracted with 300 ml of 5% ethanol by boiling for 15 min. After filtration, the filtrate was then evaporated and stored at-20 ^o^C for further analysis.

**Determination of total phenolics content**

Folin Ciocalteu reagent was used for analysis of total phenolics content (18). Briefly, 0.5 ml of the extract was mixed with 0.5 ml of Folin-Ciocalteu reagent. The solution was kept at 25^o^C for 5-8 min before adding 2 ml of sodium carbonate solution 7.5 % and adjusting the volume to 8 ml with water. After 2 h, the absorbance was measured at 725 nm. Gallic acid was used as standard for the calibration curve. Total phenolic content was expressed as mg gallic acid equivalents per gram of sample (mg/g).

**Determination of total flavonoids content**

The total flavonoid content was measured by a colorimetric assay (19). One hundred micro liters of extract was added to 4 ml of distilled water. Then, 0.3 ml 5% sodium nitrite was added. After 5 min, 0.3 ml of 10% aluminium chloride was added. In 6 min, 2 ml of 1 M sodium hydroxide was added to the mixture. Immediately, the mixture was diluted by the addition of 3.3 ml distilled water and mixed thoroughly. The absorbance was determined at 510 nm versus a blank. Catechin was used as standard for the calibration curve. Total flavonoids content of the extract was expressed as mg catechin equivalents per gram of sample (mg/g).

**DPPH radical scavenging activity**

The free radical scavenging activity of *Stinging nettle* was measured by using diphenyl-picrylhydrazyl (DPPH) assay. 5 ml of 80 mM DPPH radical solution was added to 1 ml of the *Stinging nettle* extract solutions ranging from 0.375 to 3 mg/ ml. The reaction was allowed for 30 min and absorbance was measured at 515 nm using a spectrophotometer (Rayto, China). IC50 value, the concentration of sample required to scavenge 50% of DPPH free radical, was calculated from the plotted graph of radical scavenging activity against the concentration of extracts.

**Reducing power assay**

The reducing power assay of the extract was measured according to the method used by Dharmishtha et al. (20). One milliliter of different concentrations (0.375, 0.75, 1.5 and 3 mg/ ml) of extract was mixed with 2.5 ml of phosphate buffer (200 mM; pH 6.6) and 2.5 ml of potassium hexacyanoferrate 1% and incubated at 50^o^C for 20 min. Then, 2.5 ml of 10% TCA was added to the mixture and centrifuged at 3000 rpm for 10 min. 2.5 ml of supernatant was mixed with 2.5 ml of distilled water and 0.5 ml of FeCl3 (0.1%) and the absorbance was measured at 700 nm. The results were compared with ascorbic acid which was used as a positive control. Reducing power assay of the extract was expressed as µg ascorbic acid equivalents per gram of sample (µg/g).

**Cell lines**

The Hela and BT-474 cell lines were purchased from Pasteur institute, Tehran, Iran. The cell lines were cultured in RPMI-1640 medium (PAA, Austria) supplemented with 10%fetal bovine serum and 1% antibiotics (penicillin/ streptomycin (Invitrogen)) in a humidified atmosphere containing 5% CO2 and 95% air, at 37°C.

**Cell proliferation assay**

For proliferation experiment, Hela and BT-474 cells were seed on a 96-wells microplate at a concentration of 6×10^3^ cells per well and incubated for 24 h to maximize cell attachment. Then, media were replaced with fresh media containing different concentrations (0.375, 0.75, 1.5 and 3 mg/ ml) of the *Stinging nettle* extract and cultured for 24, 48 and 72 h. After incubation, the medium was discarded and washed with phosphate buffer solution (PBS), then 50 µl of MTT stock solution (5mg/ ml) was added and incubated for 4 h at 37°C following which resulted crystals were dissolved in acidic isopropanol. After complete dissolving of formazan blue, cell proliferation was measured at 570 nm using a microplate reader (Rayto, China).

## Results

**Total phenolics content**

**Fig. 1:**
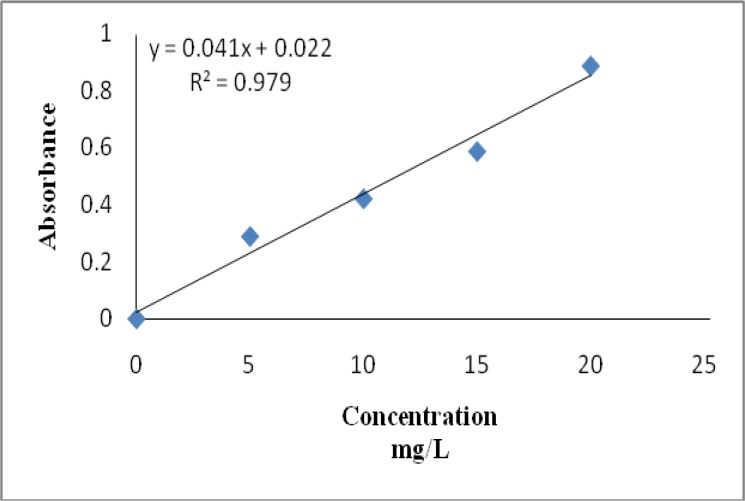
Calibration curve of gallic acid. Each point represents the mean of three experiments

Total phenolic content was estimated by gallic acid (Figure 1) and expressed as mg gallic acid equivalent (GAE)/g of extract. Table 1 represents the analytical data for phenolics content of the aqueous extract of *Stinging nettle*. Data clearly show a considerable amount of total phenolic content in aqueous extract of *Stinging nettle*.

**Total flavonoids content**

**Fig. 2:**
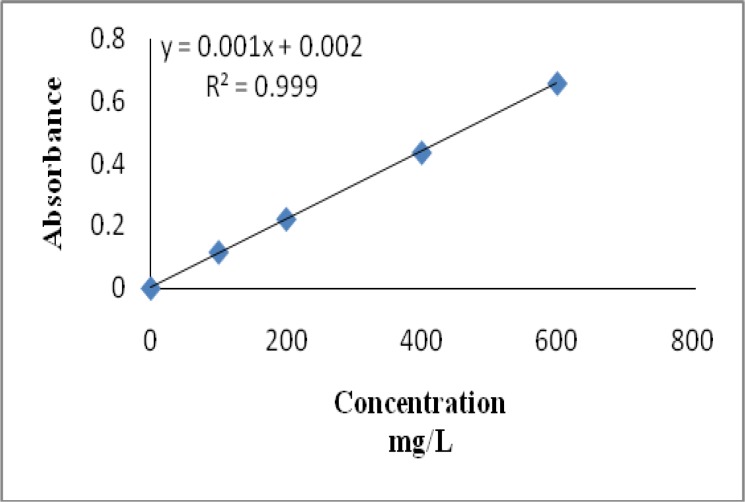
Calibration curve of Catechin. Each point represents the mean of three experiments

Total flavonoids content of aqueous extract of *Stinging nettle* was expressed as mg Catechin equivalents/ g of extract (Figure 2). Samples were analyzed in triplicate. Table 1 represents the analytical data for flavonoid content of the aqueous extract of *Stinging nettle*.

**DPPH radical scavenging activity**

The DPPH radical-scavenging capacity in the studies was reported after 30 min reaction time. The parameter used to measure the radical scavenging activity of extract evaluated is IC50 value, defined as the concentration of antioxidant required for 50% scavenging of DPPH radicals in this specified time period. The IC50 value for aqueous extract *Stinging nettle* was 1.2 mg/ ml, which was comparatively lower than the IC50 (124 μM or 21.8µg/ ml) of ascorbic acid.

**Reducing power assay**

The presence of antioxidants in the sample would result in the reduction of ferri cyanide Fe^3+^ to ferro cyanide Fe^2+^ by donating an electron. The amount of Fe^2+^ complex can then be monitored by measuring the formation of Perl's Prussian blue at 700 nm. The reducing power of extract was expressed as μg ascorbic acid equivalent per gram of extract and the results are shown in Table 1.

**Table 1:** Total phenolic, flavonoid contents and antioxidant activities of aqueous extract of *Nettle*

**Total phenolic** **mg GAE/g extract**	**Total flavonoids** **mg** **Catechin/g extract**	**Reducing power** **μg ascorbic acid/g extract**	**IC50 of DPPH radical** **(mg/ml)**
*322.941± 11.811*	133.916 ± 12.006	218.9 ± 15.582	1.2	

**Cytotoxic effect of** ***Stinging nettle*** **on Hela and BT-474 cells**

**Fig. 3:**
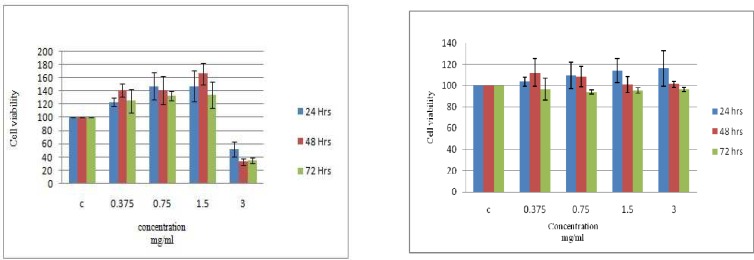
Cytotoxic effect of aqueous extracts on BT-474 (A) and HeLa (B) cells. The results are expressed as mean (SD), n = 3. The experiment was performed in triplicate

The cytotoxic effect of the aqueous extract of *Stinging nettle* was determined on BT- 474 and Hela cell lines after 24, 48 and 72 h exposure. As shown in Figure 3, cell viability of BT-474 cells was not affected by extract concentrations up to 1.5 mg/ ml but decreased to less than half of the control (no added extract) at the presence of 3 mg/ ml extract (Fig 3A). No significant changes were detected for Hela cells even in the presence of 3 mg/ ml of extract after 3 days (Fig 3B). There was no significant difference in the percentage of surviving cells between (Days 1-3) consecutive days for both BT-474 and Hela cells (P> 0.05).

## Discussion

In recent years, the use of herbal products in disease treatment has received increasing attentions due to their diverse phytometabolic contents with various chemical structures and biological activities. *Stinging nettle* has been long and widely used in folk medicine to treat various diseases. Several studies have shown multiple biological activities of *Stinging nettle* (21-24).

Plants possess high amounts of polyphenols and flavonoids and potent antioxidant activity leading therefore to various defensive and disease fighting properties. Phenolic compounds are plants secondary metabolites considered as very important plant constituents due to the presence of one or more hydroxyl groups on their aromatic ring. Those phenolic compounds being non harmful to human's health, there is an increase of the use of plants with high phenolics amount in the food industry aiming to improve the quality of foods (25).

Previous reports indicated a direct association between the amount of polyphenols present in fruits and vegetables and their cancer prevention ability (11-13). In this study we investigated the amount of total phenolics and flavonoids contents and radical scavenging activity of leaf extract of *Stinging nettle* together with its cytotoxicity effect on breast cancer and cervical cancer cell lines. The antioxidant property of *Stinging nettle* aqueous extract was also measured by reducing power, 2, 2-diphenyl-1-picrylhydrazyl (DPPH) scavenging activity method. In comparison to a previous study performed by Zoran et al. (26), our results demonstrated the presence of approximately equal amounts of phenolics content but very high flavonoids content which could be due to different standards used for measuring flavonoids. The amount of phenolics content of the aqueous extract was approximately more than 10-fold higher than methanol, chloroform, diethyl ether, ethyl acetate and butanol extracts (27). According to our data, at the concentration of 3 mg/ ml, the aqueous extract of *Stinging nettle* leaf induced a statistically significant antiproliferative effect on BT-474 cells after 24, 48 and 72 hrs (P<0.05). But no significant inhibition of cell proliferation was observed for Hela cells even after 3 days. Booth et al. showed that of 46 medicinal plant extracts, 20% were not active against HeLa cells (28). Konrad et al., showed that the grow reduction of MCF-7 cells following 5 day exposure to 1-6 mg/ ml methanolic extract from *Stinging nettle* was only 30% (29). While the leaf and root overlap slightly in their activities, each of these parts of the plant have some unique effects. Thus, according to the type of cancer, different parts of the plant may be efficient for the treatment. For example, *Urtica dioica* agglutinin, a specific lectin from *Stinging nettle* root is widely used in prostate cancer treatment (29-33). Also, it was demonstrated that the leaf of *Stinging nettle* can decrease MCF-7 cell line proliferation (17, 34). Therefore there is not always a direct relationship between the amounts of antioxidant components such as flavonoids and phenolics with cancer treatment efficiency. This may also be due to differences in the amounts of various cell surface receptors or signal transduction constituents within each cell type. Our findings also demonstrate that depending on the type of cancer cell line, certain parts of the plant may be efficient. Consequently, it is worth to examine the cytotoxic effect of extracts belonging to different parts of each plant before deepening such studies by fractionation of extract constituents.

## Conflict of interest

The authors declared no conflict of interest.
